# A comparative study of plant volatiles induced by insect and gastropod herbivory

**DOI:** 10.1038/s41598-021-02801-2

**Published:** 2021-12-08

**Authors:** Leslie Mann, Diane Laplanche, Ted C. J. Turlings, Gaylord A. Desurmont

**Affiliations:** 1 Institute of Biology, University of Neuchâtel, 11 Rue Emile-argand, 2000 Neuchâtel, Switzerland; 2 University of Canterbury, Christchurch, New Zealand; 3 European Biological Control Laboratory (EBCL USDA ARS), Montferrier-sur-lez, France

**Keywords:** Herbivory, Chemical ecology

## Abstract

Insect and gastropod herbivores are major plant consumers and their importance in the evolution of plant defensive traits is broadly recognized. However, their respective effects on plant responses have rarely been compared. Here we focused on plant volatile emissions (VOCs) following herbivory and compared the effects of herbivory by caterpillars of the generalist insect *Spodoptera littoralis* and by generalist slugs of the genus *Arion* on the VOCs emissions of 14 cultivated plant species. Results revealed that plants consistently produced higher amounts of volatiles and responded more specifically to caterpillar than to slug herbivory. Specifically, plants released on average 6.0 times more VOCs (total), 8.9 times more green leaf volatiles, 4.2 times more terpenoids, 6.0 times more aromatic hydrocarbons, and 5.7 times more other VOCs in response to 1 cm^2^ of insect damage than to 1 cm^2^ of slug damage. Interestingly, four of the plant species tested produced a distinct blend of volatiles following insect damage but not slug damage. These findings may result from different chemical elicitors or from physical differences in herbivory by the two herbivores. This study is an important step toward a more inclusive view of plant responses to different types of herbivores.

## Introduction

Plants responses to herbivory form a cornerstone of modern theories on the evolution of plant defenses^1–4^. Insects have been extensively used as models of herbivores in studies of plant defenses, with great success^5–7^. In comparison, herbivory by terrestrial gastropods (snails and slugs) has been relatively little studied^8–10^, and a major knowledge gap still exists in our understanding of how plants perceive and respond to gastropod herbivory, and how these responses play a role in multitrophic networks^11,12^.

Compared to insect herbivores, which range from extreme generalists to monophagous species, terrestrial gastropods are most often generalist and opportunistic herbivores^13^. Many species do feed on live plant material but are not necessarily obligate herbivores and include other types of food (decaying organic matter, seeds, live or dead animals) to their diet^11,13,14^. They also tend to show highly dynamic foraging patterns^15^ and do not stay on the same plant over extended periods of time, which can make them elusive to study. These characteristics do not mean that they do not impact plant fitness: snails and slugs are considered important pests of a variety of cultivated plants^16^. They are major consumers of plant seedlings in nature and can have great effects on species composition and succession in plant communities^17–19^. These effects have been shown to be mediated by plant secondary metabolites in several systems^20,21^, pointing at the importance of herbivorous gastropods as selective forces for plant defensive traits. Recently, it was shown that herbivory by *Arion* slugs decreases the production of volatile organic compounds (VOCs) in *Brassica rapa* plants infested by a lepidopteran caterpillar, consequently decreasing plant attractiveness to natural enemies^11^. This prompted a broader investigation of how plants respond to gastropod herbivory compared to insect herbivory in terms of volatile emissions.

A wide variety of VOCs are produced by plants, either constitutively or induced in response to different stresses. The volatiles produced in response to herbivore damage are commonly referred to as herbivore induced-plant volatiles (HIPVs). These volatiles are divided in different categories depending on their chemical structure and biosynthetic pathway. The most commonly accepted categories of plants volatiles are fatty acid derivatives, terpenes and phenylpropanoids/benzenoids^22,23^. Among fatty acid derivatives, green leaf volatiles (GLVs) are of particular importance: they are rapidly released by plants as a general “wound response” when green tissue is damaged but can also be released in specific amounts or ratios in response to herbivory^24–26^. Several ecological functions have been proposed regarding the role of plant volatiles in nature. Namely, leaf volatiles have been hypothesized to have evolved to reduce oxidative stress, as a means of within-plant signaling or between-plant signaling, and as a means to attract natural enemies that may help reduce insect damage, the latter being often referred to as a “cry for help”^27–29^. Experimental tests of these hypotheses have often been carried out focusing on insect herbivores. Using gastropods as models in such studies could provide a different perspective about the evolution and ecology of plant volatiles and plant–herbivore interactions in general.

Here we measured and compared volatile emissions of leaves attacked by caterpillars or slugs in 14 cultivated species from nine plant families. We selected this large spectrum of plant species to capture general trends of volatile emissions in response to these herbivores. We used caterpillars of the generalist noctuid *Spodoptera littoralis* as insect herbivore, and the slug *Arion vulgaris* as gastropod herbivore. Based on the above-mentioned observation that slugs may suppress inducible plant volatiles^11^, we formulated the following hypotheses: (i) plant volatile emissions in response to caterpillar and slug herbivory differ qualitatively and quantitatively; (ii) patterns of volatile emissions in response to caterpillar and slug herbivory are plant-specific; (iii) plant volatile emissions are lower in response to slug herbivory compared to caterpillar herbivory. To test the first two hypotheses, the volatile emissions of control plants and plants damaged by the two herbivores were collected and analyzed using gas chromatography and mass spectrometry (GC–MS). The total quantities of VOCs produced were compared, and differences between the whole blends of VOCs emitted were investigated by running principal component analyses (PCAs) with the main chemical compounds found for every plant species tested. In order to test the third hypothesis, caterpillar and slug damage on leaf tissues was quantified, and the quantities of VOCs released per cm^2^ of leaf tissue damage were compared.

## Material and methods

### Insect and slug material

Slugs were collected in natural areas of France (Beaune) and Switzerland (Neuchâtel, La Tène, Thielle, and Fribourg) and were maintained in groups of 20 in clear plastic boxes (25 × 15 × 27 cm) containing autoclaved soil (Classic Profisubstrat, Einheitserde, Germany). They were fed once a week with store-bought cabbages, carrots, mushrooms, raw pasta and dog food (Mini Menu Matzinger, Purina). The rearing boxes were kept in a climate chamber set at a constant temperature of 12 °C, 80% air humidity, and a 14/8 (L:D) photoperiod. It is very challenging to identify slugs from the genus *Arion* to the species level based on external morphology alone, and several species occur in the areas where they were collected, principally *A. vulgaris*, *A. ater* and *A. rufus*. Thus, a molecular analysis via PCR of the eggs laid by some of the slugs collected for the study was performed, and results showed that all the eggs belonged to the species *Arion vulgaris* (results not shown).

*Spodoptera littoralis* eggs were provided by Syngenta (Stein, Switzerland) on a weekly basis. The eggs were kept in clear plastic boxes (13 × 15 × 5 cm) at 25 °C until hatching. After egg hatch, the young larvae were stored in a climate chamber set at a temperature of 12 °C and a 14/8 (L–D) photoperiod. The larvae were fed artificial diet (F9219B, Frontier Scientific Services, Newark, USA). We used 4 to 8 days old larvae in the experiments.

### Plant material

The following 14 plant species were tested in this study: beetroot (*Beta vulgaris* L.), cauliflower (*Brassica oleracea botrytis* L.), Chinese cabbage (*Brassica rapa* var. *pekinensis* L.), turnip (*Brassica rapa var. rapa* L.), cucumber (*Cucumis sativus* L.), artichoke (*Cynara scolymus* L.), carrot (*Daucus carota* L.), fennel (*Foeniculum vulgare* Mill), sunflower (*Helianthus annuus* L.), coco bean (*Phaseolus vulgaris* L.), rhubarb (*Rheum rhabarbarum* L.), tomato (*Solanum lycopersicum* L.), wheat (*Triticum aestivum* L.), and maize (*Zea mays* var. *Delprim* L.). The seeds were purchased from Mauser Samen (Samen Mauser AG., Winterthur, Switzerland) and Select (Wyss Samen une Pflanzen AG., Zuchwill, Switzerland) (Table 1). The seeds of each plant species were placed in separate cylindrical plastic boxes (8.5 × 5 cm) on a 2 cm layer of humidified glass beads for germination, at room temperature (approx. 20 °C) and natural photoperiod. After germination, the sprouts were planted in planting soil (Classic Profisubstrat, Einheitserde, Germany), in individual plastic tubes (4 × 11 cm). They were then placed in a greenhouse under natural light conditions and ambient temperatures (ranging from 15 to 35 °C). Plants were used for the experiments after 4 to 6 weeks of growth.

**Table 1:** List of the plant species used in the study.

Species name	Plant family	Common name	VOCs collection	Damage measurements	Ratio VOCs/cm^2^ damage
*Beta vulgaris*	Amaranthaceae	Beetroot	✓	✓	✓
*Brassica oleracea botrytis*	Brassicaceae	Cauliflower	✓	✓	✓
*Brassica rapa* var. *pekinensis*	Brassicaceae	Chinese cabbage	✓	✓	✓
*Brassica rapa* var. *rapa*	Brassicaceae	Turnip	✓	✓	✓
*Cucumis sativus*	Cucurbitaceae	Cucumber	✓	✓	✓
*Cynara scolymus*	Asteraceae	Artichoke	✓	✓	✓
*Daucus carota*	Apiaceae	Carrot	✓	×	×
*Foeniculum vulgare*	Apiaceae	Fennel	✓	×	×
*Helianthus annuus*	Asteraceae	Sunflower	✓	✓	✓
*Phaseolus vulgaris*	Fabaceae	Coco bean	✓	✓	✓
*Rheum rhabarbarum*	Polygonaceae	Rhubarb	✓	✓	✓
*Solanum lycopersicum*	Solanaceae	Tomato	✓	✓	✓
*Triticum aestivum*	Poaceae	Wheat	✓	✓	✓
*Zea mays*	Poaceae	Maize	✓	✓	✓

Check marks (✓) show the different collections/measures (VOCs collection, damage measurements, and calculation ratio VOCs/cm^2^ damage) that were performed with each species.

All protocols using plants and animals in this study complied with relevant institutional, national, and international guidelines and legislations.

### Volatile collections

For each plant species, 15 plants were selected and randomly divided into three treatments: control plants with no herbivores (n = 5), plants infested by *S. littoralis* (n = 5), and plants infested by *A. vulgaris* (n = 5). In total, VOCs were collected from 240 plants over a three-week period. The day before VOCs collections, early in the afternoon (ca. 1:30 PM), the plants were individually placed in transparent nalophan bags (Nalophan, Omya AG, Oftringen, Switzerland), which were closed with masking tape, Parafilm (Bemis Company, USA) and rubber bands. In order to inject clean air into the bags and to collect the plant volatiles, two hollow cylindrical glass tubes (1 × 3 cm) were placed on the inside of each bag, on opposite sides of the plant. They were capped on the outside of the bag by open screw caps, safely securing the plastic bag trapped in-between. A hole was then pierced through the plastic, in the middle of the locked tube and cap, allowing for the insertion of the air inlet in one tube, and the volatile filter connected to the air outlet in the other tube^30^. Herbivores were randomly placed on leaves before closing the bags. For slug-infested plants, 3 slugs were used per plant. In order to standardize slug size, the slugs were weighed beforehand and slugs belonging to the 5–10 g range were used. For caterpillar-infested plants, 20 *S. littoralis* first instar larvae were used per plant. Plants were left overnight under greenhouse light conditions (natural sunlight supplemented in the morning with neon lights between 7 and 13 h), and volatiles were collected in the morning, leaving the herbivores in the bags. Plants were connected to the volatile collection device^31^ for 2:30 h with a purified air entrance flow of 0.9 L per minute (LPM) and an exit flow of 0.8 LPM. The collection filters contained 25 mg of 80–100 mesh superQ absorbent (Sigma, Buchs, Switzerland). Before use, collection filters were cleaned with 500 μL of methylene chloride (HPLC grade). After each collection, VOCs were eluted from the filters with 100 μL of methylene chloride. A solution of two pure compounds, n-octane and nonyl acetate, acting as internal standards was added to each sample (200 ng of each compound). The samples were then stored in a freezer at − 80 °C until the GC–MS analyses. Once the volatile collections were completed, the leaves of all plants were excised and scanned, enabling us to record herbivore damage as a measure of (i) leaf area eaten and (ii) percentage of the total leaf area eaten using the Adobe Photoshop software (Adobe, San Jose, USA).

VOCs were analyzed with an Agilent 6890 gas chromatograph, which was coupled to a 5973 Network mass selective detector (transfer line 230 °C, source 230 °C, ionization potential 70 eV). A 2 μL aliquot of each sample was injected in pulsed splitless mode onto a non-polar column (HP-1 ms, 30 m, 0.25 mm ID, 0.25 μm film thickness, Agilent J&W Scientific, USA). Helium at constant flow (1.9 mL/min) was used as carrier gas. After injection, the temperature was maintained at 40 °C for 3 min, then increased to 100 °C at 8 °C/min, and then to 220 °C, at 5 °C/min. The quantities of the major components of the blends (ca. 10–15 compounds per blend) were roughly estimated based on the peak areas of the compounds compared to the peak areas of the internal standards. Compounds were identified by comparing the spectra obtained from the samples with those from a reference database (NIST mass spectral library). Not all identifications could be confirmed by comparing the spectra of the samples to pure reference compounds. Compounds were classified in one of the following categories in accordance with their chemical structure: green leaf volatiles (GLVs), terpenoids, aromatic hydrocarbons, alkanes/alkenes, aldehydes, other (VOCs that did not belong to one of the abovementioned categories), and unknown (VOCs that could not be identified). For the statistical analyses, the compounds belonging to the categories alkanes/alkenes, aldehydes, other, and unknown were pooled in one category “sum of other VOCs”. For two plant species (fennel and carrot), herbivore damage could not be measured because the leaves of these two plants were too feathery/narrow: although traces of damage were visible on the plants, the surface eaten could not be clearly defined and measured. For these two plant species, the ratio VOCs emitted/cm^2^ of leaf damage could therefore not be calculated (Table 1).

### Statistical analyses

The total amounts of VOCs (i.e. sum of the relative quantities of individual compounds compared to internal standards) produced by plants, as well as the amounts of VOCs belonging to different classes of compounds (GLVs, terpenoids, aromatic hydrocarbons, and sum of other VOCs), the total amount of damage caused by herbivores (cm^2^), the percentage of leaf area consumed by slugs and caterpillars, and the ratio of VOCs emitted per cm^2^ of damage were compared using parametric 1-way ANOVA after a quadratic root transformation of the data in order to meet the assumptions of the model, and means were compared using the Tukey post-hoc procedure (α = 0.05). The association between the total amount of VOCs and the damaged area (cm^2^) was explored for each plant and herbivore species using correlation tests (Pearson's method, α = 0.05).

The complete blends of volatiles produced by control plants and plants damaged by slugs and caterpillars were compared using non-discriminant principal component analysis (PCA) for each plant species tested. For each sample, every volatile compound received a score of 0 (absent) or 1 (present), and the dataset created with these scores was used to run the PCAs. We then used the projections of the two first principal components and the 90% confidence ellipses of each treatment (control, infested by slug, infested by caterpillar) to visually assess the pattern of response of the plants to slug and caterpillar damage. If the ellipses of two treatments largely overlapped, we considered that the plants responded similarly to the two treatments. If there was a small overlap or no overlap at all, we considered that the responses diverged. The compounds included in the PCA analyses were the main compounds that were consistently produced by each plant species in response to each treatment. The number of compounds included in the blends ranged from 8 compounds (turnip) to 26 compounds (tomato). Minor compounds and compounds that were not consistently produced (i.e. only present in ≤ two samples) were excluded from the analyses. All statistical procedures were performed using the JMP15 statistical software (JMP® 15, SAS Institute Inc., Cary, NC, https://www.jmp.com/home.html).

## Results

### Patterns of volatile emissions in response to caterpillar and slug herbivory

**Figure 1:**
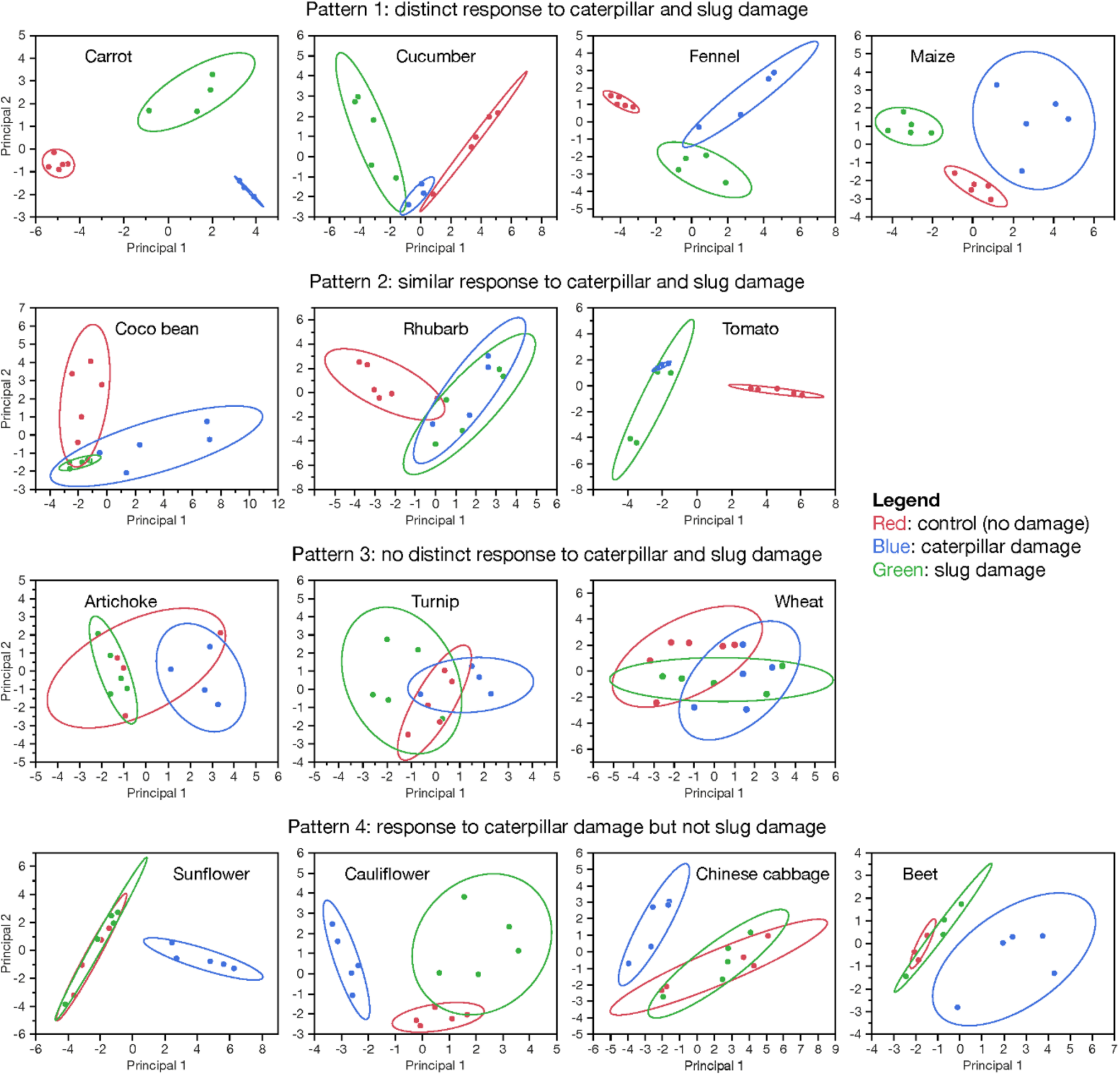
Principal components analyses of all plant species showing the four patterns of VOCs emissions observed for control (undamaged) plants, caterpillar-damaged plants, and slug-damaged plants. Pattern 1: distinct response to caterpillar and slug damage. Pattern 2: similar response to caterpillar and slug damage. Pattern 3: no response pattern to caterpillar or slug damage. Pattern 4: Response to caterpillar damage but not to slug damage. For each graph, the horizontal and vertical axes show projections on to the first and second principal components, respectively. Each dot represents a sample (= 1 plant) JMP® 15.

The PCAs of the blends of volatiles produced by control plants and plants damaged by caterpillar and slug herbivores revealed four patterns of volatile responses among the 14 plant species: (1) highly distinct responses to caterpillar and slug damage. The blends of VOCs produced in response to caterpillar and slug damage did not overlap with each other and did not overlap with VOCs produced by control plants (or very little). This pattern was observed with four species: cucumber, fennel, carrot, and maize. (2) Similar responses to slug and caterpillar damage. The blends of VOCs produced in response to caterpillar and slug damage largely overlapped with each other but did not overlap with VOCs produced by control plants (or very little). This pattern was observed with three species: tomato, coco bean, and rhubarb. (3) No response pattern to either caterpillar or slug damage. The blends of VOCs produced by control plants and plants damaged by caterpillar and slug herbivores largely overlapped with each other. This pattern was observed with three plant species: artichoke, turnip, and wheat. (4) Response to caterpillar damage but not to slug damage. The blend of VOCs produced in response to caterpillar damage did not overlap with the blend emitted by control plants, whereas the blend produced in response to slug damage largely overlapped with the blend emitted by control plants. This pattern was observed with four plant species: beet, cauliflower, Chinese cabbage, and sunflower. A fifth pattern could have been theoretically observed: response to slug damage but not to caterpillar damage. This pattern was not observed for any of the 14 species tested. The PCAs results of all 14 species are shown in Fig. 1. All the compounds identified in the volatile emissions of each species are listed in Supplementary Table 1.

### Quantitative emissions of VOCs and amounts of leaf damage caused by caterpillar and slug herbivory

**Figure 2:**
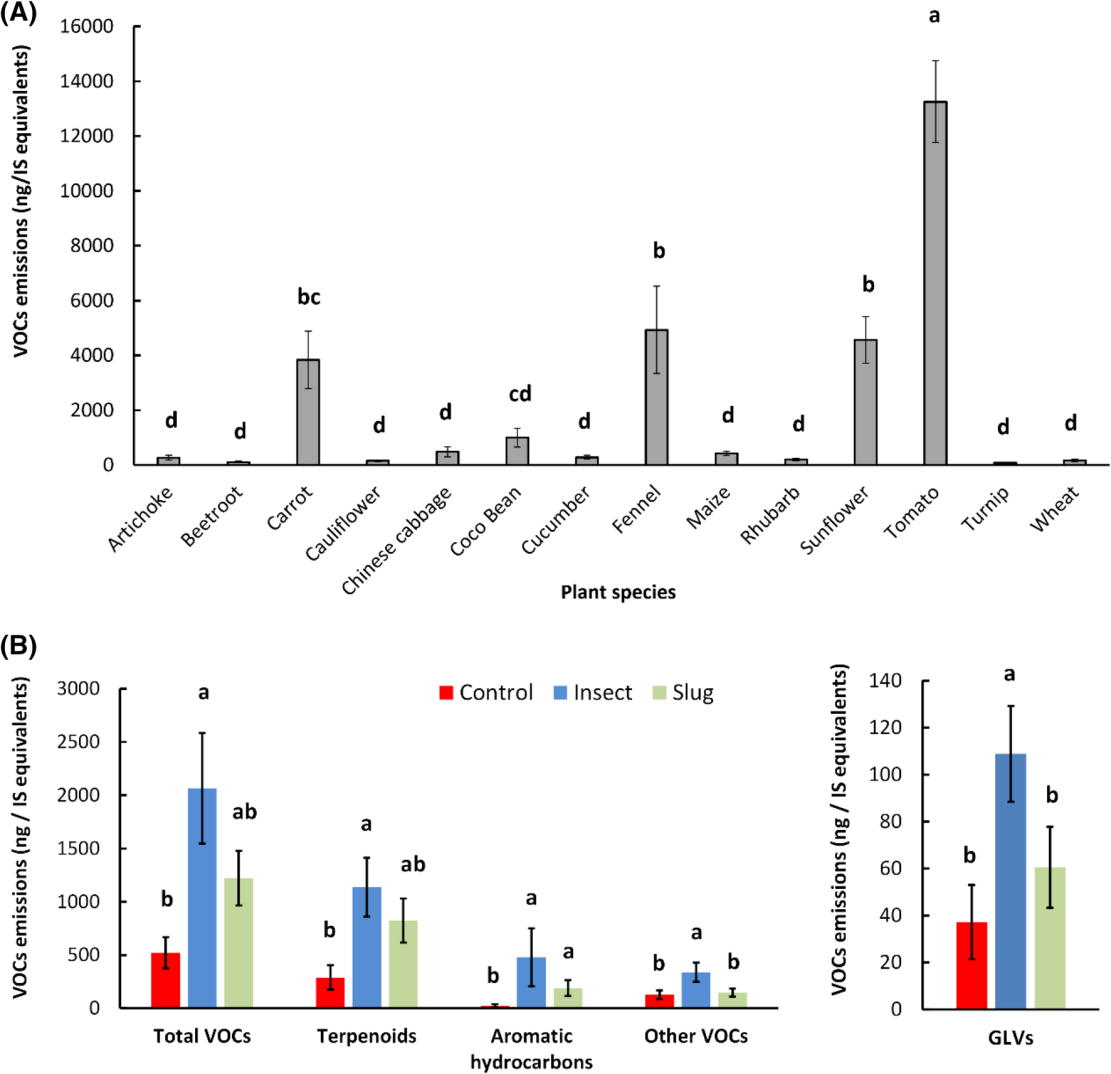
Variation in volatile emissions depending on plant species and herbivory treatment. (**A**) Total VOCs emissions per species, all herbivory treatments (control, caterpillar-damaged, slug-damaged) included. (**B**) VOCs emissions [Total VOCs, Terpenoids, Aromatic hydrocarbons, Green leaf volatiles (GLVs), and other VOCs] depending on herbivory treatment for all species excluding tomato. Means with a different letter are statistically different (One-way ANOVA, Tukey post-hoc test, α = 0.05, JMP® 15).

There was tremendous variation in volatile emissions among the plant species included in the study. The total amounts of VOCs emitted per species, independently of herbivory treatment, differed significantly among species (F_13,196_ = 34.6, P < 0.0001): tomato plants, by far, produced the highest quantities of VOCs, followed by sunflower, fennel, and carrot. All other species produced much lower amounts of VOCs (Fig. 2A). Because tomato volatile emissions were considerably larger than all other species and because the experimental setup may have affected tomato volatile emissions (see “Discussion”), analyses on the effects of herbivory treatment (control, caterpillar-damaged, and slug-damaged) on volatile emissions were performed including and excluding tomato from the analyses. When tomato plant was excluded, there was a difference in the total quantity of VOCs emitted by control, caterpillar-damaged, and slug-damaged plants (F_2,192_ = 5.8, P = 0.003): caterpillar-damaged plants emitted more volatiles than control plants and slug-damaged plants emitted intermediate levels of volatiles. A similar pattern was observed for terpenoid emissions (F_2,192_ = 3.9, P = 0.02). Caterpillar-damaged plants also produced more GLVs than control and slug-damaged plants (F_2,192_ = 11.7, P < 0.0001). Emissions of aromatic hydrocarbons were higher for caterpillar-damaged and slug-damaged plants than for control plants (F_2,192_ = 4.5, P = 0.01). Finally, emissions of other VOCs were higher for caterpillar-damaged plants than slug-damaged and control plants (F_2,192_ = 6.2, P = 0.002). When tomato was included in the analysis, the differences in terpenoids emissions (F_2,207_ = 2.46, P = 0.09), aromatic hydrocarbons emissions (F_2,207_ = 3.0, P = 0.054), and other VOCs (F_2,207_ = 2.5, P = 0.08) became non-significant, but the differences in total quantity of VOCs emitted (F_2,207_ = 3.1, P = 0.047) and GLVs (F_2,207_ = 10.6, P < 0.0001) remained significant. Results are summarized in Fig. 2B, and species-specific data are available in Supplementary Table 2.

**Figure 3:**
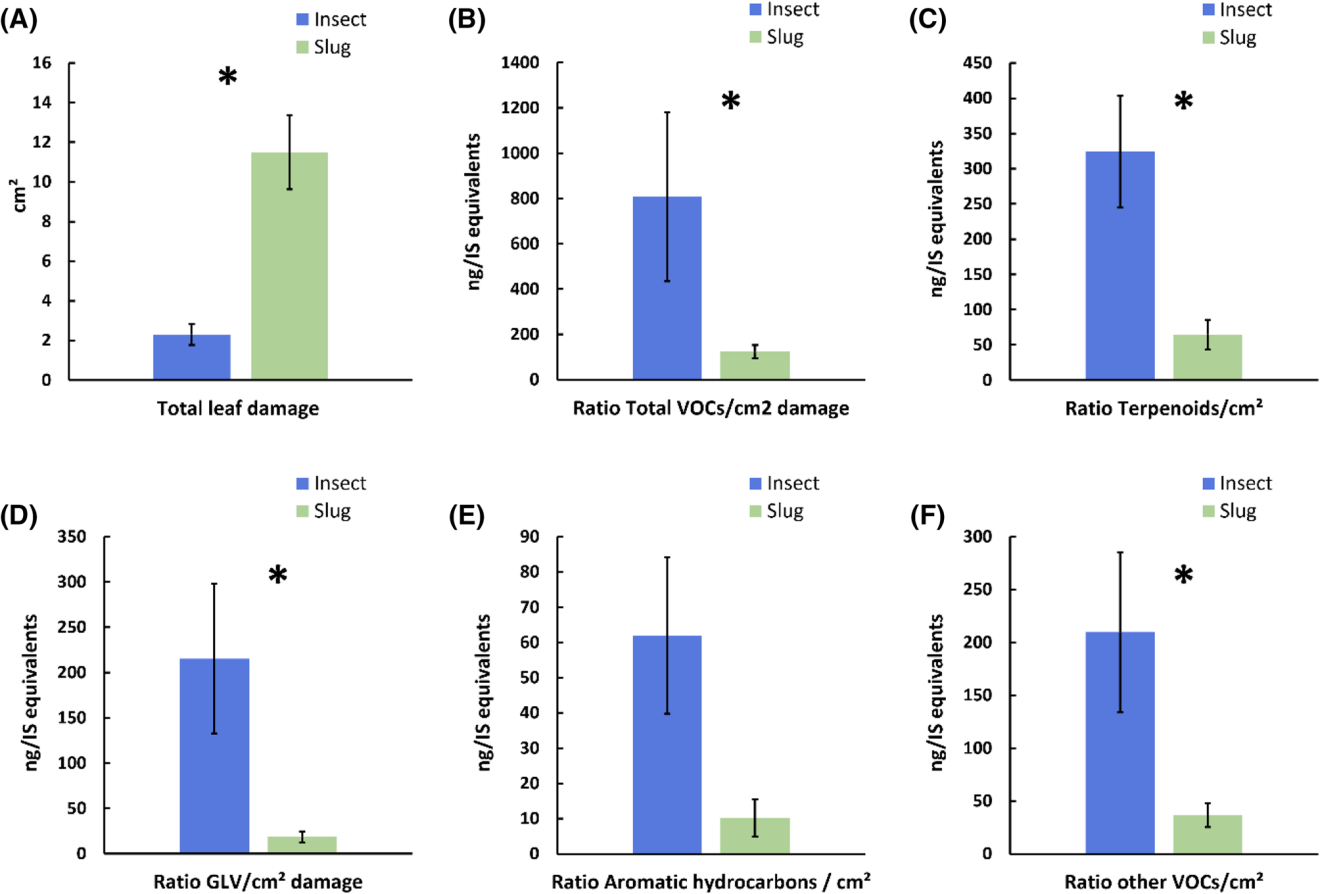
Volatile emissions (ng/IS equivalents) and leaf area consumed (cm^2^) for all plant species damaged by a caterpillar herbivore (*Spodoptera littoralis*) and a slug herbivore (*Arion vulgaris*) (mean ± SE). (**A**) Total leaf damage (i.e. leaf area eaten); (**B**) Ratio total VOCs emissions/cm^2^ of leaf damage; (**C**) Ratio terpenoids/cm^2^ of leaf damage; (**D**) Ratio green leaf volatiles (GLV) emissions/cm^2^ of leaf damage; (**E**) Ratio aromatic hydrocarbons/cm^2^; (**F**) Ratio other VOCs/cm^2^ of leaf damage. Means with an asterisk are statistically different (One-way ANOVA, α = 0.05, JMP® 15).

Overall, slugs consumed five times more leaf tissue than caterpillars, when comparing damage on all plant species tested, including tomato (F_1,117_ = 57.3, P < 0.0001). On average, slugs consumed 5.7 ± 1.0% of the total leaf surface, and caterpillars consumed only 1.4 ± 0.3% of the total leaf surface (F_1,117_ = 58.0, P < 0.0001). The ratio of VOCs emitted per cm^2^ leaf damage was in general significantly higher in response to caterpillar damage than to slug damage. This was true for the total VOCs emitted/cm^2^ (F_1,117_ = 28.2, P < 0.0001), GLVs emitted/cm^2^ (F_1,117_ = 19.5, P < 0.0001), terpenoids emitted/cm^2^ (F_1,117_ = 12.1, P = 0.007), and the sum of other VOCs (F_1,117_ = 32.3, P < 0.0001), but not for aromatic hydrocarbons emitted/cm^2^ (F_1,117_ = 3.1, P = 0.08) (Fig. 3). When excluding tomato from the analysis, all these results remained similar.

Correlation tests (Pearson's method) between the total number of VOCs and the damaged area (cm2) for each plant and herbivore species gave high positive correlations for turnip and artichoke with the insect treatment (r = 0.938, P = 0.018, and r = 0.866, P = 0.058, respectively). These high positive correlations mean that the more the plant was damaged, the higher amounts of VOCs were produced. Other correlations were non-significant (Supplementary Table 3); low sample numbers (5 replications per plant/herbivore pair) reduced the power of these tests.

## Discussion

Plant responses to herbivory have been largely studied using insects as models of herbivores, neglecting other groups of plant consumers such as terrestrial gastropods. The current study was conducted as a step toward filling the gap in our understanding of how plants perceive and respond to herbivory by snails and slugs. We chose to focus on a specific type of plant response, the production of volatile compounds in response to leaf damage, and compared the effects of slug and caterpillar herbivory on volatile emissions across a range of 14 cultivated species from nine plant families. The general conclusion of the study is that plants produce in general much lower amounts of VOCs in response to slug herbivory than in response to caterpillar herbivory, and that plant response to slug herbivory is often unspecific: for 50% of the plants tested, the blend of volatiles released by slug-damaged plants did not qualitatively differ from the blend emitted by control plants.

The hypotheses that (i) plant emissions in response to caterpillar and slug herbivory vary qualitatively and quantitatively, and (ii) patterns of volatile emissions in response to caterpillar and slug herbivory are plant-specific, were supported by the results of the PCAs analyses, which allowed whole-blend comparisons between herbivory treatments for each plant species tested. Four patterns of response to herbivory were observed (Fig. 1) and were almost evenly distributed among the species tested: four species showed a distinct response pattern to both slug and caterpillar herbivory (pattern 1), three showed the same response pattern to slug and caterpillar herbivory (pattern 2), three showed no distinct pattern of response to either slug or caterpillar herbivory (pattern 3), and four showed a distinct response to caterpillar damage but no response to slug damage (pattern 4). In other words, 11 out of the 14 plant species emitted a blend of volatiles that differed from control plants in response to caterpillar damage (patterns 1, 2, and 4), but only 7 emitted a blend of volatiles that differed from control plants in response to slug damage (patterns 1 and 2). The plants that did not respond to caterpillar or slugs (pattern 3) were among the species that emitted the lowest amounts of VOCs: turnip, wheat, and artichoke (Figs. 1 and 2A, table S2). Conversely, species that emitted clearly distinguishable volatile blends were often species that emitted high amounts of VOCs in general (e.g.: fennel, carrot, tomato) (Figs. 1 and 2A). Patterns of responses did not seem to strongly depend on plant family: the four plant families that had at least two species represented in the study (Brassicaceae, Asteraceae, Apiaceae, and Poaceae) showed different patterns of response within the family, with the exception of the two species belonging to the Apiaceae family (carrot and fennel), which both exhibited pattern 1 (Fig. 1). Interestingly, the theoretical pattern “response to slug damage, but no response to caterpillar damage” was never observed.

Tomato was an outlier among the species tested: tomato plants released much higher amounts of volatiles than all other species, and even control (undamaged) plants released high amounts of terpenoids and other VOCs. Qualitatively, this species still emitted a distinct blend of volatiles in response to herbivory (pattern 2), but there was no discernable increase in the amounts of VOCs produced after herbivory (Table S2). It is possible that the volatile collection setting (plants were placed in individual nalophan bags 20 h prior to volatile collections) stressed the plants and induced the release of GLVs^32^, which are known to trigger the production of terpenes in tomatoes^33^. Damage to leaf trichomes during plant handling may also have accounted for the exceptionally high emission rates from tomato plants^34^. Stress responses associated with the setting may have also affected the volatile emissions of other plant species^35^, but less drastically.

The hypothesis that (iii) plant volatile emissions are reduced in response to slug herbivory compared to caterpillar herbivory was supported by the quantitative analyses of VOCs. Even though slugs consumed on average five times more leaf tissue than the caterpillars, plants produced in general more VOCs after caterpillar damage than after slug damage (Fig. 2B). When standardized by cm^2^ of leaf damage, the difference between the amounts of VOCs released after caterpillar and slug herbivory became even more significant: plants released on average 6.0 times more VOCs (total), 8.9 times more GLVs, 4.2 times more terpenoids, 6.0 times more aromatic hydrocarbons, and 5.7 times more other VOCs in response to 1 cm^2^ of caterpillar damage than in response to 1 cm^2^ of slug damage (Fig. 3).

Why do plants respond less quantitatively and with less specificity to slug herbivory than to insect herbivory? It would be tempting to seek an adaptive explanation and posit that there is an evolutionary benefit to produce VOCs after caterpillar herbivory that does not apply to slug herbivory. This hypothesis would be particularly interesting to explore in the context on the “cry for help” ecological function of plant volatiles^36^, which proposes that HIPVs are produced to recruit natural enemies that fend off the plant attackers. Plants damaged by insects can “call” plenty of reliable airborne natural enemies (predators and parasitoids) which can pick up VOCs in the atmosphere and arrive to attack herbivores^28^. However, most natural enemies of slugs and snails are predators foraging on the ground (e.g. carabid beetles), and their use of plant volatiles when foraging has rarely been shown^37^. Additionally, due to their highly dynamic foraging patterns, gastropods are likely to be gone from the plant attacked once the VOCs have been released and perceived by natural enemies. Therefore, there may be no benefit in crying for help through airborne volatiles for a plant damaged by gastropods.

Induction of specific defensive responses in plants depends on the presence of chemical elicitors associated with herbivore damage^38,39^. The oral secretions of caterpillars are known to contains several of these elicitors^40^. Slugs may be stealthier feeders and possibly have ways to avoid the induction of plant defenses or even to suppress them. A study by Kästner et al.^10^ showed that the mucus of the slug *Deroceras reticulatum* contains significant amounts of salicylic acid. This common plant defense hormone is typically involved in the regulation of plant defenses against pathogens and may, through cross talk, suppress defenses against herbivores, which is typically regulated by jasmonic acid^41,42^. The notion that inducible plant defenses, including HIPVs, may not work against slugs or may be manipulated by slugs warrants further research.

Despite being generalist herbivores, *A. vulgaris* and *S. littoralis* show preferences for certain host plants. Members of the Brassicaceae and Apiaceae families are in general highly palatable to *A. vulgaris*, while members of the Ranunculaceae and Poaceae are less palatable, although high variability can be observed within each family^43^. Clear preferences are also known for *S. littoralis*, with plant species such as clover, maize, cotton, and tomato consistently receiving more damage and/or being preferred for oviposition^44,45^. However, no detectable association between herbivore preference in nature and volatile emissions was observed: plant species belonging to the preferred species or families of either *A. vulgaris* or *S. littoralis* did not consistently exhibit a specific pattern of volatile emissions in the PCA analyses.

Evolutionary hypotheses regarding plant responses to herbivory should preferentially be tested with wild plants and under realistic conditions. The plants included in our study were all cultivated varieties and artificial selection may have changed their VOCs emissions compared to wild populations^46^. Turnip (*Brassica rapa*), for example, was the lowest emitting plant from all species tested in our study and did not show any pattern of response after herbivory by either caterpillar or slug. However, wild populations of *B. rapa* are known to emit complex and specific volatile blends in response to different types of herbivores^47,48^. Similar differences have been observed for cabbage when comparing wild and domesticated populations^49^.

Differences in VOCs profiles and amounts produced in response to caterpillars and slugs could also be a consequence of the feeding patterns of the two herbivores^50^. Young *S. littoralis* caterpillars consistently made multiple small holes in the leaf tissue, often attacking the youngest and most tender leaves. Slugs, on the other hand, ate larger chunks of leaf material. Young leaves are in general known to produce higher amounts of VOCs than mature leaves^51^. When comparing HIPVs from maize plants induced by different species of *Spodoptera* caterpillars, de Lange et al. found that *S. frugiperda* induced lower amounts of VOCs. One of the proposed explanations was that it takes larger bites than the other species, but this did not fully explain the observed differences^52^. The temporal patterns of feeding could also have played a role: volatiles were collected 20 h after placing the herbivores on the plants, and it is not known when the damage on the leaves precisely occurred. Temporal dynamics of feeding can play an important role in the blend of VOCs released by a plant^53^. For certain plants like cotton^54^, some inducible emissions take more than 24 h after feeding damage before being emitted. It is therefore possible that some of these late emitted volatiles were missed in our study. Finally, separating herbivore-induced plant volatiles from the volatiles associated with the herbivores themselves may also help explaining some of the patterns observed, although caterpillars and slugs typically release very low amounts of volatiles compared to damaged plants^11,48^.

The present study was designed to explore general trends of volatile emissions across a wide range of plant families and species. This approach reduced our power to perform in-depth analyses at the species level. Various volatile compounds collected during the study remained unidentified. Moreover, the analyses were only performed with the main compounds emitted to reduce the overall amount of noise inherent to volatolomic datasets^55^, and some minor but important compounds may have been overlooked. This study represents a first step toward an integrated view of plant responses to herbivores that includes terrestrial gastropods. The finding that, in general, plant response to slug herbivory is less complex and specific than insect herbivory invites to further examination in a proper ecological and evolutionary framework^56^. Plenty is left to explore on the slimy side of plant–herbivore interactions.

## Supplementary Information

Supplementary Information.
