# Supplementary material for: A comparative study of plant volatiles induced by insect and gastropod herbivory
Source: Sci Rep. 2021 Dec 8;11:23698. doi: 10.1038/s41598-021-02801-2 (PMC8654843; doi:10.1038/s41598-021-02801-2)
Supplement: Supplementary file 1 — Supplementary Information. [file 41598_2021_2801_MOESM1_ESM.pdf]

Supplementary data

Supplementary table 1. Volatile organic compounds produced by the 14 plant species used in the study.

Supplementary table 2. Species-specific differences in herbivore damage and quantitative volatile emissions for every plant species tested (mean ± SE).

Supplementary table 3. Correlations between the leaf area damaged and the quantity of VOCs produced for each pair herbivore/plant species.

Supplementary table 1. Volatile organic compounds produced by the 14 plant species used in the study

**Compound**	**Cucumber**	**Chinese cabbage**	**Cauliflower**	**Turnip**	**Beetroot**	**Tomato**	**Sunflower**	**Bean**	**Fennel**	**Carrot**	**Wheat**	**Maize**	**Rhubarb**	**Artichoke**
**GREEN LEAF VOLATILE**														
3-3Hexen-1-ol	X												X	
Butanoic acid, 3-hexenyl ester (3-Hexen-1-ol)								X						
Cis-3-hexen-1-ol (Fluka)					X									
(Z)-3-hexen-1-ol (Fluka)		X					X	X				X		X
Z-3-Hexenol							X							
Z-3-Hexenyl acetate		X	X	X	X		X	X	X	X	X	X		X
**TERPENE**														
2-Carene		X				X	X	X			X			
3-Carene						X	X		X	X		X	X	
Allo-ocimene									X					
Alpha-farnesene	X	X	X	X	X			X	X	X			X	
Alpha-humulene (Fluka)		X				X								
Alpha-phellandrene			X			X								
Alpha-pinene	X	X	X			X	X		X	X				
Alpha-selinene			X											
Alpha-terpinene or terpilene			X			X	X			X				
Alpha-thujene			X											
Aristolene or calarene							X							
Beta-bergamotene (maize)									X	X		X	X	
Beta-bisabolene										X				
Beta-bourbonene							X							
Beta-caryophyllene		X									X			
Beta-elemene			X				X							
Beta-farnesene								X		X		X		
Beta-myrcene			X			X	X		X	X		X		
Beta-pinene										X				
Beta-phellandrene						X								
Beta-terpinene	X		X											
Beta-thujene	X		X											

Borneol							X							
Camphene	X						X		X	X				
Carene											X	X		
Carene undecane		X												
Caryophyllene							X	X	X					
Cis-ocimene									X	X				
Citral	X					X		X						
Cyclohexene						X						X		
Estragole									X					
Eucalyptol			X											
Fenchone									X					
Germacrene							X		X	X				
R(+)-Limonene		X	X				X		X	X		X	X	
Linalool (maize)												X		
Phenanthrene		X												
Sabinene							X						X	
Squalene	X													
Sylvestrene	X		X				X	X				X	X	
Trans-caryophyllene (Fluka)						X				X				X
Trans-ocimene	X		X			X	X	X		X	X		X	
Tridecatetraene = (E,E)-4,8,12-tri methyl-1,3,12-trimethyl-1,3,7,11-tridecatetraene							X							
**ALKANE OR ALKENE**														
Cyclohexane 1,2,3 dimethyl	X													
Cyclopentane, 1,2,3,4,5-pentamethyl				X				X						X
Cyclopentene						X								
Cyclopentene, 1,2,3,3,4-pentamethyl								X			X	X		
Dodecane	X	X					X						X	
Heptane, 2,2,4,6,6-pentamethyl-		X											X	
Hexadecane	X												X	
Nonatriene				X								X	X	X
Pentadecane (Fluka)		X									X			

Tetradecane													X	
Tridecane	X													
Undecane							X							
**AROMATIC HYDROCARBON**														
Anisole									X					
Benzoic acid		X						X				X		
Beta-cymene						X	X	X						
Indole (Fluka)	X							X				X		
O-cymene						X								
P-cymene		X				X								
Pyridine, 2-fluoro-Cyclohexane, 1-methyl-2-propyl-				X										
Veratrole										X				
**ALDEHYDE**														
Decanal					X			X					X	
Nonanal	X	X	X	X	X		X	X			X	X	X	X
**CLASSIFIED AS OTHER**														
1,7-Octadien-3-one, 2-methyl-6-met 22849 041702-60-7 49 hylene-		X												
2-Methyl-2-hepten-6-on (aldrich)	X													
Acetic acid, 1,7,7-trimethyl-bicyclo[2.2.1]hept-2-yl ester							X							
Cyclobutene, bis(1-methylethylidene)						X								
Isothiocyanic acid				X										
Octenol								X						
Sulfur-containing compound 1								X				X		X
Sulfur-containing compound 2			X								X			
**UNKNOWN**														
Unknown 5.2	X													
Unknown 5.5					X							X		X
Unknown 7.4												X		
Unknown 8.2	X	X									X			

Unknown 8.26								X						
Unkown 8.9		X												
unknown 9.95										X				
Unknown 10.64											X			
Unknown 10.7								X			X			
Unknown 10.98											X		X	
Unknown 11.9		X												
Unknown 12.2								X						
Unknown 12.6			X		X						X			
Unknown 13.47													X	
Unknown 13.85						X								
Unknown 14						X								
Unknown 16.7							X							
Unknown 22.66										X				

Supplementary file 2: herbivore damage (cm²) and volatile emissions (ng / IS equivalents) for each plant species (mean ± SE).

Plant species	Measure	Treatment		
Artichoke		Control	Insect	Slug
	Total Vocs	320.65 ±176.17	200.02 ±50.03	300.68 ±192.96
	GLV	274.66 ±175.20	66.75 ±18.48	241.34 ±176.37
	Terpenes	3.97 ±3.97	44.88 ±22.02	8.91 ±8.91
	Alkanes or alkenes	10.70 ±2.91	34.85 ±13.19	11.10 ±3.02
	Aromatic hydrocarbons	0.00 ±0.00	0.00 ±0.00	0.00 ±0.00
	Aldehydes	9.04 ±5.50	7.61 ±5.13	7.88 ±3.93
	Others	4.56 ±1.96	6.04 ±1.83	7.52 ±2.07
	Unknowns	21.70 ±8.93	30.97 ±6.91	32.85 ±24.46
	Damaged leaf area	NA	1.20 ±0.40	3.80 ±1.95
	Percentage of leaf eaten	NA	0.72 ±0.26	2.47 ±1.26
Beetroot		Control	Insect	Slug
	Total Vocs	54.46 ±16.13	217.46 ±27.55	65.73 ±23.35
	GLV	32.94 ±16.07	130.34 ±20.60	44.52 ±22.37
	Terpenes	0.00 ±0.00	6.04 ±4.36	0.00 ±0.00
	Alkanes or alkenes	6.68 ±1.12	9.25 ±1.86	5.98 ±2.85
	Aromatic hydrocarbons	0.00 ±0.00	0.00 ±0.00	0.00 ±0.00
	Aldehydes	1.17 ±1.17	6.48 ±4.68	7.19 ±3.37
	Others	6.73 ±1.98	5.82 ±1.65	4.68 ±1.94
	Unknowns	6.95 ±4.30	59.53 ±11.75	3.36 ±2.09
	Damaged leaf area	NA	0.32 ±0.08	7.40 ±1.41
	Percentage of leaf eaten	NA	0.35 ±0.10	7.15 ±1.53
Carrot		Control	Insect	Slug
	Total Vocs	54.46 ±16.13	7340.39 ±2091.69	4061.13 ±731.08
	GLV	32.94 ±16.07	219.35 ±106.82	28.54 ±17.89
	Terpenes	0.00 ±0.00	4346.97 ±1424.94	3312.49 ±493.94
	Alkanes or alkenes	6.68 ±1.12	0.00 ±0.00	0.00 ±0.00
	Aromatic hydrocarbons	0.00 ±0.00	453.78 ±185.23	122.83 ±53.74
	Aldehydes	1.17 ±1.17	0.00 ±0.00	0.00 ±0.00
	Others	6.73 ±1.98	0.00 ±0.00	0.00 ±0.00
	Unknowns	6.95 ±4.30	2320.29 ±484.63	597.27 ±349.42
	Damaged leaf area	NA	NA	NA
	Percentage of leaf eaten	NA	NA	NA
Cauliflower		Control	Insect	Slug
	Total Vocs	207.23 ±65.83	102.84 ±51.51	150.87 ±33.61
	GLV	0.00 ±0.00	10.82 ±8.77	5.36 ±4.25
	Terpenes	165.66 ±55.49	73.36 ±43.63	115.76 ±31.18
	Alkanes or alkenes	4.81 ±1.99	1.23 ±1.23	8.70 ±4.82
	Aromatic hydrocarbons	0.00 ±0.00	0.00 ±0.00	0.00 ±0.00

	Aldehydes	15.93 ±9.36	3.20 ±2.16	2.74 ±1.68
	Others	7.04 ±0.51	4.06 ±1.69	6.28 ±1.60
	Unknowns	13.78 ±3.08	10.17 ±4.45	12.03 ±3.90
	Damaged leaf area	NA	0.93 ±0.43	26.82 ±18.56
	Percentage of leaf eaten	NA	0.68 ±0.35	6.42 ±1.13
Chinese cabbage		Control	Insect	Slug
	Total Vocs	894.94 ±526.44	285.18 ±51.76	275.56 ±123.89
	GLV	11.02 ±6.14	119.13 ±50.00	6.48 ±4.56
	Terpenes	810.67 ±512.62	97.55 ±27.58	177.36 ±131.60
	Alkanes or alkenes	7.66 ±3.73	11.91 ±5.58	9.16 ±4.70
	Aromatic hydrocarbons	10.81 ±6.52	19.81 ±9.95	36.50 ±30.58
	Aldehydes	2.54 ±1.68	3.25 ±2.00	6.20 ±4.43
	Others	5.17 ±2.67	7.40 ±3.20	5.04 ±3.39
	Unknowns	32.22 ±2.86	26.14 ±5.83	30.85 ±7.99
	Damaged leaf area	NA	10.98 ±3.99	40.06 ±13.14
	Percentage of leaf eaten	NA	4.00 ±1.50	14.15 ±3.62
Coco bean		Control	Insect	Slug
	Total Vocs	1507.42 ±982.02	846.26 ±243.69	636.59 ±255.18
	GLV	0.00 ±17.01	323.65 ±143.83	101.39 ±35.78
	Terpenes	91.54 ±39.04	206.08 ±46.40	271.48 ±85.69
	Alkanes or alkenes	11.75 ±2.25	16.04 ±4.32	7.95 ±1.35
	Aromatic hydrocarbons	17.11 ±10.17	77.22 ±27.26	30.15 ±12.87
	Aldehydes	4.18 ±2.69	11.75 ±9.05	4.09 ±2.56
	Others	17.22 ±11.64	8.04 ±2.44	12.62 ±4.48
	Unknowns	788.37 ±426.35	177.34 ±75.76	201.96 ±134.82
	Damaged leaf area	NA	1.26 ±0.38	19.29 ±3.88
	Percentage of leaf eaten	NA	0.47 ±0.13	7.61 ±1.42
Cucumber		Control	Insect	Slug
	Total Vocs	155.06 ±53.39	221.75 ±75.16	471.56 ±178.34
	GLV	2.25 ±1.46	2.65 ±2.65	11.88 ±5.41
	Terpenes	70.06 ±25.72	154.30 ±66.60	388.32 ±145.37
	Alkanes or alkenes	31.21 ±14.33	8.31 ±1.53	8.00 ±2.43
	Aromatic hydrocarbons	0.00 ±0.00	1.32 ±1.32	35.87 ±21.97
	Aldehydes	20.91 ±15.08	10.37 ±4.89	6.88 ±1.88
	Others	7.54 ±4.09	2.47 ±1.58	1.07 ±1.07
	Unknowns	23.08 ±3.49	42.33 ±20.13	19.54 ±6.72
	Damaged leaf area	NA	1.69 ±0.93	10.97 ±1.28
	Percentage of leaf eaten	NA	0.97 ±0.43	8.44 ±1.79
Fennel		Control	Insect	Slug
	Total Vocs	567.15 ±262.37	10539.60 ±3639.06	3686.56 ±416.54
	GLV	31.60 ±25.37	0.00 ±0.00	0.00 ±0.00
	Terpenes	285.03 ±174.85	5235.38 ±1306.82	1501.01 ±346.89
	Alkanes or alkenes	0.00 ±0.00	0.00 ±0.00	0.00 ±0.00
	Aromatic hydrocarbons	250.42 ±183.19	5263.24 ±3002.22	2172.27 ±290.75

	Aldehydes	0.00 ±0.00	0.00 ±0.00	0.00 ±0.00
	Others	0.00 ±0.00	0.00 ±0.00	0.00 ±0.00
	Unknowns	0.10 ±0.10	40.98 ±14.06	13.28 ±5.76
	Damaged leaf area	NA	NA	NA
	Percentage of leaf eaten	NA	NA	NA
Maize		Control	Insect	Slug
	Total Vocs	250.58 ±61.12	652.04 ±162.79	372.35 ±143.33
	GLV	20.79 ±12.73	98.86 ±38.06	50.17 ±29.67
	Terpenes	119.28 ±29.02	357.12 ±72.76	204.28 ±86.00
	Alkanes or alkenes	19.81 ±4.15	27.34 ±7.96	14.37 ±6.97
	Aromatic hydrocarbons	39.69 ±23.61	96.04 ±40.82	41.62 ±22.03
	Aldehydes	9.14 ±1.31	7.04 ±2.17	16.75 ±11.79
	Others	5.29 ±1.38	5.60 ±1.41	6.15 ±1.59
	Unknowns	36.58 ±17.53	60.05 ±23.27	39.01 ±20.99
	Damaged leaf area	NA	0.85 ±0.09	4.19 ±1.97
	Percentage of leaf eaten	NA	0.71 ±0.08	3.03 ±1.35
Rhubarb		Control	Insect	Slug
	Total Vocs	245.74 ±97.43	232.82 ±43.52	129.93 ±55.85
	GLV	0.00 ±0.00	19.45 ±6.55	37.36 ±24.78
	Terpenes	122.42 ±83.30	64.94 ±24.34	40.22 ±24.98
	Alkanes or alkenes	34.70 ±14.15	40.25 ±23.51	8.67 ±6.69
	Aromatic hydrocarbons	0.00 ±0.00	0.00 ±0.00	0.00 ±0.00
	Aldehydes	28.54 ±19.37	28.51 ±22.82	6.62 ±5.54
	Others	0.00 ±0.00	0.00 ±0.00	0.00 ±0.00
	Unknowns	60.07 ±18.23	79.67 ±35.61	37.05 ±8.26
	Damaged leaf area	NA	0.80 ±0.18	6.40 ±2.14
	Percentage of leaf eaten	NA	0.93 ±0.36	4.01 ±1.31
Sunflower		Control	Insect	Slug
	Total Vocs	2285.72 ±1335.45	5955.03 ±1218.19	5454.13 ±1555.10
	GLV	61.60 ±61.60	363.52 ±67.45	196.76 ±84.99
	Terpenes	2015.82 ±1232.96	4104.66 ±658.22	4635.13 ±1379.08
	Alkanes or alkenes	0.00 ±0.00	51.86 ±25.56	0.00 ±0.00
	Aromatic hydrocarbons	0.00 ±0.00	307.97 ±288.99	14.41 ±9.82
	Aldehydes	62.04 ±28.11	904.23 ±210.13	442.68 ±146.58
	Others	30.12 ±30.12	19.12 ±11.71	20.64 ±12.64
	Unknowns	116.15 ±28.20	203.66 ±97.05	144.52 ±51.90
	Damaged leaf area	NA	4.01 ±0.61	14.68 ±3.69
	Percentage of leaf eaten	NA	2.03 ±0.23	12.35 ±3.39
Tomato		Control	Insect	Slug
	Total Vocs	17149.24 ±1957.26	11188.91 ±1542.41	11412.66 ±3407.18
	GLV	0.00 ±0.00	0.00 ±0.00	0.00 ±0.00
	Terpenes	12014.10 ±2455.42	10701.21 ±1525.71	10926.96 ±3208.15
	Alkanes or alkenes	3955.68 ±2466.99	1.84 ±1.84	0.00 ±0.00
	Aromatic hydrocarbons	962.92 ±110.30	449.90 ±26.16	439.50 ±165.54

	Aldehydes	0.00 ±0.00	0.00 ±0.00	0.00 ±0.00
	Others	18.26 ±7.90	3.78 ±3.78	0.00 ±0.00
	Unknowns	198.28 ±75.40	32.18 ±21.08	46.20 ±38.17
	Damaged leaf area	NA	1.85 ±0.59	7.21 ±2.34
	Percentage of leaf eaten	NA	1.05 ±0.25	4.58 ±1.30
Turnip		Control	Insect	Slug
	Total Vocs	38.37 ±14.24	102.41 ±25.16	101.10 ±63.47
	GLV	8.67 ±6.35	55.08 ±18.18	58.87 ±52.16
	Terpenes	5.05 ±3.20	23.11 ±11.50	6.58 ±4.60
	Alkanes or alkenes	9.72 ±3.02	13.00 ±2.04	10.37 ±3.56
	Aromatic hydrocarbons	4.23 ±1.75	4.07 ±1.77	7.14 ±2.20
	Aldehydes	9.51 ±7.89	4.22 ±2.64	5.39 ±3.53
	Others	0.00 ±0.00	0.87 ±0.87	6.31 ±4.35
	Unknowns	1.18 ±1.18	2.06 ±1.34	6.43 ±4.06
	Damaged leaf area	NA	0.79 ±0.20	8.77 ±3.23
	Percentage of leaf eaten	NA	0.60 ±0.13	4.93 ±1.78
Wheat		Control	Insect	Slug
	Total Vocs	207.63 ±134.82	142.37 ±32.76	168.08 ±87.01
	GLV	0.00 ±0.00	4.96 ±3.31	3.72 ±2.33
	Terpenes	40.58 ±30.75	59.95 ±24.01	45.61 ±19.42
	Alkanes or alkenes	5.45 ±2.62	16.45 ±7.81	2.80 ±1.75
	Aromatic hydrocarbons	0.00 ±0.00	0.00 ±0.00	0.00 ±0.00
	Aldehydes	3.86 ±1.76	7.58 ±3.33	1.76 ±1.11
	Others	7.26 ±1.93	5.71 ±2.82	6.59 ±0.46
	Unknowns	157.74 ±106.16	53.43 ±20.19	114.18 ±65.46
	Damaged leaf area	NA	2.52 ±1.26	2.10 ±1.00
	Percentage of leaf eaten	NA	2.48 ±1.03	2.84 ±1.33

Supplementary file 3: correlations between herbivore damage (cm²) and total VOCs emissions (ng / IS equivalents) for each plant species (mean ± SE).

Correlation tests (Pearson's method). Values are comprised between -1 and +1. Positive values indicate a positive correlation and negative values indicate a negative correlation. P-values < 0.05 indicate statistically significant correlations.

Species	Total VOCs / damaged area (cm2)			
	Herbivore treatment	Correlation (r)	P-value	
Artichoke	Insect	0.86567	0.0579	.
	Slug	0.59335	0.2916	
Beetroot	Insect	0.5528	0.3339	
	Slug	0.71707	0.1728	
Cauliflower	Insect	-0.3456	0.5689	
	Slug	-0.0222	0.9717	
Chinese cabbage	Insect	-0.4502	0.4467	
	Slug	-0.0819	0.8959	
Coco bean	Insect	0.0746	0.9051	
	Slug	0.26904	0.6616	
Cuccumber	Insect	-0.5847	0.3005	
	Slug	0.39678	0.5084	
Maize	Insect	0.47924	0.414	
	Slug	0.32028	0.5993	
Rhubarb	Insect	-0.0846	0.8924	
	Slug	0.25666	0.6768	
Sunflower	Insect	0.51623	0.3732	
	Slug	-0.5963	0.2885	
Tomatoe	Insect	0.18662	0.7638	
	Slug	-0.2722	0.6577	
Turnip	Insect	0.93819	0.01827	*
	Slug	0.80326	0.1016	
Wheat	Insect	0.35066	0.5629	
	Slug	-0.2591	0.6739	
